# Epidemiology and Outcome of Ventilator-Associated Pneumonia in a Heterogeneous ICU Population in Qatar

**DOI:** 10.1155/2016/8231787

**Published:** 2016-06-13

**Authors:** Husain Shabbir Ali, Fahmi Yousef Khan, Saibu George, Nissar Shaikh, Jameela Al-Ajmi

**Affiliations:** ^1^Department of Medical ICU, Hamad General Hospital, P.O. Box 3050, Doha, Qatar; ^2^Department of Internal Medicine, Hamad General Hospital, P.O. Box 3050, Doha, Qatar; ^3^Department of Surgical ICU, Hamad General Hospital, P.O. Box 3050, Doha, Qatar; ^4^Department of Infectious Disease, Hamad General Hospital, P.O. Box 3050, Doha, Qatar

## Abstract

*Objective*. The purpose of this study is to collect data on epidemiology, microbiology, and outcome of VAP in our ICUs for reevaluation of the therapeutic strategies.* Methods*. This retrospective study involved all adult patients, 15 years of age or older, diagnosed with VAP in multidisciplinary ICUs at Hamad General Hospital between January 2010 and December 2012.* Results*. A total of 106 patients were enrolled. The mean incidence of VAP was 5.0 per 1000 ventilator-days. It was predominant among younger age group (<60 years), male patients (80.2%), and trauma ICU admissions (49.0%). The most common comorbidity was hypertension (34%) and polytrauma (36.8%) was the most frequent admission diagnosis. 30-day mortality was 23.6% and it was significantly higher in ≥60 years age group, female gender, patients with diabetes mellitus, hypertension, chronic respiratory disease, ≥1 comorbidity, and poor functional status, smokers, medical and surgical ICU admissions, and patients with previous stay in medical/surgical wards, inappropriate empirical therapy, and admission diagnosis of respiratory failure. Gram-negative bacilli were the most frequent respiratory specimen isolates,* Pseudomonas spp.* being the most common. Majority of our* Acinetobacter* isolates were multidrug resistant.* Conclusion*. The incidence of VAP in our ICUs was low. Higher mortality rates were observed in certain subgroup of patients. Resistance to commonly used antimicrobials is likely to require reevaluation of the therapeutic strategies at our institution.

## 1. Introduction

Despite advances in preventive strategies, diagnostic techniques, and treatment modalities, ventilator-associated pneumonia (VAP) remains the most common infectious complication among patients admitted in intensive care units (ICUs). It results in high morbidity and mortality, prolonged lengths, and increased cost of hospitalization. The incidence of VAP varies widely in different studies depending on the diagnostic criteria used, type of ICU, and patient population. Moreover, the causative organisms vary according to the patients' demographics in the ICU, the duration of hospital/ICU stay, and the antibiotic policy of the institution [1]. Therefore, incidence of VAP and the associated microbial flora needs to be studied in local setting so as to allow more effective utilization of antimicrobial agents [2]. This prompted us to conduct a study, to describe the epidemiology, causative organisms, and outcome of VAP in a heterogeneous ICU population at a tertiary care center in Doha, State of Qatar, from January 2010 to December 2012.

## 2. Materials and Methods

### 2.1. Study Design and Setting

This retrospective study included all adult patients (15 years of age or older) clinically diagnosed with VAP in multidisciplinary ICUs at Hamad General Hospital, Qatar, between January 2010 and December 2012. Hamad General Hospital is a 603-bed tertiary care center that covers all specialties except for haematology-oncology, cardiology, and obstetrics. It has been Joint Commission International (JCI) accredited since 2006 and is the first hospital system in the region to achieve institutional accreditation from the Accreditation Council for Graduate Medical Education-International (ACGME-I). There are three adult ICUs in Hamad General Hospital, namely, medical ICU (MICU) with 22 beds, surgical ICU (SICU) with 12 beds, and trauma ICU (TICU) with 15 beds.

### 2.2. Definitions

Ventilator-associated pneumonia (VAP) was defined as per Center of Disease Control (CDC) as a pneumonia that occurs in a patient who has been intubated and ventilated for two or more calendar days on the date of the event. Clinical diagnosis of pneumonia was defined as the presence of a new or progressive pulmonary infiltrates or consolidation or cavitation in chest radiography, associated with at least two of the following criteria: body temperature of >38°C or <36°C with no other known cause, leucocytes count <4000/mm^3^ or >12000/mm^3^, and purulent tracheal secretion or a change in characteristics of an existing secretion [3]. All patients clinically diagnosed with VAP had respiratory specimen collected for Gram stain and culture-sensitivity, which was either deep tracheal aspirate or bronchoalveolar lavage (BAL).

VAP rates were described in accordance with the standard established by the National Control System of Nosocomial Infection of the Centers for Disease Control and Prevention (rate = number of VAP cases/1000 mechanical ventilator-days) [4]. Comorbidities were defined by case records of patients. Clinical severity was assessed by Sequential Organ Failure Assessment (SOFA) score [5, 6].

The following pathogens were considered as MDR (multidrug resistant):* methicillin-resistant Staphylococcus aureus (MRSA)*, extended-spectrum *β*-lactamase producing Gram-negative* Enterobacteriaceae* (*ESBL*),* Pseudomonas aeruginosa*, and other nonfermenting organisms (*Acinetobacter baumannii, Stenotrophomonas maltophilia*) resistant to three or more of the following antibiotic classes: antipseudomonal cephalosporins or penicillins, carbapenems, fluoroquinolones, and aminoglycosides [7]. Empirical antimicrobial therapy was considered as appropriate when initiated no later than 48 hours after VAP diagnosis and when it included at least one antimicrobial agent to which the etiological agent was described as susceptible in the antibiogram result [8].

### 2.3. Source of Information and Data Collection

Cases were identified by Infection Control Team at Hamad General Hospital based on the above diagnostic criteria. This was followed by retrospective chart review. Data were collected on a special form which included patient demographics, clinical characteristics, laboratory results, and outcome (30-day mortality).

### 2.4. Data Analysis

Descriptive statistics in the form of mean, standard deviation for interval variables, and frequency with percentages for categorical variables were used. One-way ANOVA was performed to see significant mean differences among ICU groups and chi-square tests were performed to see significant association between ICU groups and other categorical variables. *p* value 0.05 with two-tailed test was considered statistically significant. Data analysis was performed with SPSS software (v 21.0; IBM Corp., Armonk, NY, USA).

## 3. Results

Over the 3-year study period (January 2010 to December 2012), 126 episodes of VAP were reported in three ICUs. For determination of incidence all episodes of VAP were considered. For collection of demographic, microbiological, and outcome data twenty patients were excluded: one because of age less than 15 years and the other due to having a second episode of VAP and another eighteen due to incomplete records. The remaining 106 patients with complete records had their charts reviewed.

### 3.1. Incidence and Demographics

**Figure 1:**
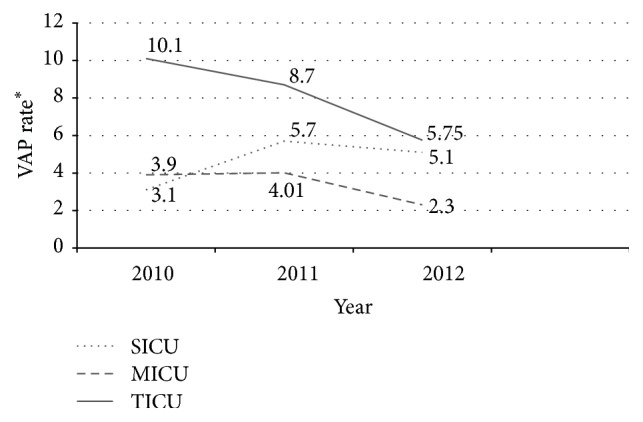
Mean ventilator-associated pneumonia (VAP) rates in SICU, MICU, and TICU during study period. VAP  rate^*∗*^ = (number of ventilator-associated pneumonias/number of ventilator-days) × 1000.

The overall mean incidence rate of VAP was 5.0 per 1000 ventilator-days and the cumulative VAP rates in each year were 5.42 per 1000 ventilator-days in 2010, 5.91 per 1000 ventilator-days in 2011, and 3.88 per 1000 ventilator-days in 2012. VAP rate ranged from as high as 10.1 per 1000 ventilator-days in TICU during 2010 to as low as 2.3 per 1000 ventilator-days in MICU during 2012.  Figure 1 shows comparison between the mean VAP rates in MICU, SICU, and TICU during the 3-year study period.

The mean age of patients was 46.6 ± 18.6 years (range 16–90 years) and 73.6% patients (78/106) were less than 60 years old. There were 80.2% (85/106) males and 29.2% (31/106) Qatari patients (Table 1).

### 3.2. Comorbidities and Associated Conditions

The most frequent comorbidity was hypertension (36/106, 34.0%), and the most frequent associated condition was smoking, while the most frequent ICU admission diagnosis was polytrauma (39/106, 36.8%). The mean Sequential Organ Failure Assessment (SOFA) score on admission was 6.5 ± 3.4 (range 1–15); it was highest among patients admitted to MICU. The duration of mechanical ventilation before diagnosis of VAP was 11.9 ± 9.5 days (range 2–46 days) and it was longest in patients admitted to medical ICU (*p* = 0.003); refer to Table 1.

### 3.3. Microbial Pattern and Antimicrobial Susceptibility

Deep tracheal aspiration was done to collect respiratory sample in 96/106 (90.6%) cases and BAL was done in the remaining 10/106 (9.4%) cases. Single organism was isolated from respiratory specimen of 52/106 (49%) patients and ≥2 organisms isolated from another 52/106 (49%) patients and cultures were negative in 2 patients. The most common isolate was* Pseudomonas* species (Table 2); 43/106 (40.6%) patients had at least one organism which was MDR and 61/106 (57.5%) had completely sensitive strains. Table 2 summarizes the antibiotic sensitivity pattern of the most frequent microorganisms isolated in this study.

### 3.4. Outcome and Comparison between Survivors and Nonsurvivors

The 30-day mortality in our study was 25/106 (23.6%). Several variables were evaluated for association with clinical outcome, as presented in Table 3. Among patients who developed VAP, mortality was significantly higher in age group ≥60 years (*p* = 0.001), female gender (*p* = 0.02), and those with ≥1 comorbidity (*p* = 0.001); in patients with diabetes mellitus (*p* = 0.012), hypertension (*p* = 0.001), and preexisting respiratory disease (*p* = 0.004). It was also observed that smokers (*p* = 0.012), bed bound patients (*p* = 0.018), MICU admissions (*p* = 0.001), patients admitted from medical/surgical wards (*p* = 0.001), patients receiving inappropriate empirical therapy (*p* = 0.001), and those having admission diagnosis of respiratory failure (*p* = 0.001) had higher mortality. Mortality in patients with sensitive organisms was 16.4% and MDR organisms was 32.6% (*p* = 0.054). Patients with respiratory specimen isolates of single organism, ≥2 organisms, and negative culture had mortality rates of 30.8%, 15.4%, and 50%, respectively. There was no significant relationship between specific microorganisms and mortality.

## 4. Discussion

Novelty of our work comes from being the first to study VAP in the State of Qatar. The mean VAP incidence in our study was 5.0 per 1000 ventilator-days, which falls below the rate of 8.8 per 1000 ventilator-days reported in European and South American ICUs [9] and is comparable to the incidence reported in other Gulf countries (4.8 per 1000 ventilator-days) [10]. The reason for our lower incidence rate is not clear. It could be due to involvement of young population with short period of ventilation or due to variability in the definition of VAP or it might reflect efficiency of the preventive strategies and critical care practices in our ICUs. VAP prevention bundle is one of the major strategies used at our institution for reducing the incidence of VAP. It comprises the following components: elevation of the head of the bed between 30 and 45 degrees, daily sedation interruption and assessment of readiness to extubate, use of subglottic suction endotracheal tubes, peptic ulcer disease prophylaxis, deep vein thrombosis prophylaxis, and oral care with chlorhexidine solution.

As noted in this series, the patients were younger (46.56 ± 18.57 years) than those reported in a retrospective cohort study using data from a large US inpatient database (61.7 ± 19.2 years) [11]. Also they were younger than patients with ICU-acquired sepsis in the European SOAP study (60.0 ± 17.4 years) [12]. The reason for this remains obscured and needs to be investigated. In agreement with many reports, our study showed male predominance [8, 11, 13] which can be explained by the fact that Qatar and other Gulf countries have large working community composed mainly of males. Male sex is one of the nonmodifiable patient-related risk factors for the development of VAP [14].

A multicenter study from Greece, involving a mixed ICU population, has reported 45% of its VAP cases to have ICU admission due to trauma (multiple injury: 29% and head injury: 16%) [13] which coincides with our findings, where we had 49% of patients from TICU. Higher incidence of VAP has been reported in patients with neurocritical illness [15]. In our study, 18.9% (20/106) of the VAP cases were initially admitted to ICU due to neurological disease. 30-day mortality in this subgroup was 40% (8/20). The SOFA score of our VAP patients on ICU admission (6.46 ± 3.36) was lower than that reported in the PneumA trial (7.3 ± 4 and 7.4 ± 4 in the 8-day and 15-day antibiotic regimen, resp.) [16]. This could be related to the difference in the indication for initial ICU admission in both studies.

In our population, Gram-negative bacteria were the most common pathogens causing VAP with* Pseudomonas aeruginosa* being the most frequent isolate (Table 2). Similar findings were reported by many authors [1, 17]. Most of the* Acinetobacter baumannii* isolates were resistant to piperacillin/tazobactam and carbapenems, which raises doubts about the ongoing efficacy of these agents in the empirical treatment of VAP at our hospital.

Mortality associated with VAP has varied in different studies depending on the term used (in-hospital mortality versus attributable mortality versus 30-day mortality), institution, and study population. The 30-day mortality in our study was 23.6%, which falls within the range of 20%–75% described in recent studies [1]. In our population, mortality was higher in the elderly subgroup (age ≥ 60 years) as shown in Table 3 and is consistent with recent reports [18]. As per our observation, females developed less VAP but experienced increased mortality (42.9% versus 18.8%, *p* = 0.02) and this confirms the results from previous studies [19]. Resende and coworkers have emphasized on the fact that the presence of comorbidities has significant impact on the outcome of patients with VAP [8]. Similar results are seen in our analysis where preexisting hypertension, diabetes mellitus, chronic respiratory disease, presence of ≥1 comorbid condition, and poor functional status have been associated with increased mortality (Table 3). In agreement with many reports, our data showed a significant association between inappropriate empirical therapy and increased mortality [2, 5, 20, 21]. This highlights the importance of local studies in providing information about the most likely causative organisms and their resistance patterns to assist clinicians in selecting appropriate empirical treatment, if VAP is suspected.

This study has several limitations. Firstly, due to its retrospective design, there was incomplete data for some factors related to mortality and compliance to VAP prevention bundles. Secondly, the study was conducted in one hospital, thereby limiting the generalization of these results to other institutions in Qatar. Thirdly, the diagnostic criteria for VAP which we used might differ from those used by other hospitals. This may lead to overestimation or underestimation of the incidence and not allow comparison with other institutions. Despite these limitations, this study is the first step in highlighting the problem of VAP in the State of Qatar, and it has implications for future work.

## 5. Conclusion

The overall incidence of VAP in our ICUs was low. Higher mortality rates were associated with increasing age, female gender, presence of comorbidities, admission to medical ICU, patients admittance from wards, primary diagnosis of respiratory failure, and inappropriate empirical therapy. Resistance to piperacillin/tazobactam and carbapenems is likely to require reevaluation of the therapeutic strategies used at Hamad General Hospital. We recommend conducting prospective multicenter studies to know local risk factors and patterns of pathogens causing VAP to assist in making appropriate preventive, diagnostic, and therapeutic plans to reduce their incidence and improve outcomes.

## Figures and Tables

**Table 1:** Clinical data of patients admitted with VAP.

Characteristic	MICU *N* = 27 (%)	SICU *N* = 27 (%)	TICU *N* = 52 (%)	Overall *N* = 106 (%)
Age (mean ± SD) (years)	57.9 ± 16.2	53.1 ± 15.9	37.3 ± 16.4	46.6 ± 18.6

Gender				
Male	17 (63)	21 (77.8)	47 (90.4)	85 (80.2)
Female	10 (37)	6 (22.2)	5 (9.6)	21 (19.8)

Ethnicity				
Qatari	10 (37)	6 (22.2)	15 (28.8)	31 (29.2)
African	2 (7.4)	5 (18.5)	7 (13.5)	14 (13.2)
Indian subcontinent	12 (44.4)	13 (48.1)	22 (42.3)	47 (44.3)
Southeast Asians	0	0	5 (9.6)	5 (4.7)
Others	3 (11.2)	3 (11.2)	3 (5.8)	9 (8.5)

Comorbidities				
Diabetes mellitus	12 (44.4)	11 (40.7)	7 (13.5)	30 (28.3)
Hypertension	15 (55.6)	13 (48.1)	8 (15.4)	36 (34)
Cardiovascular disease	6 (22.2)	6 (22.2)	3 (5.8)	15 (14.2)
Renal disease	3 (11.1)	2 (7.4)	2 (3.8)	7 (6.6)
Neurological condition	6 (22.2)	1 (3.7)	0	7 (6.6)
Respiratory disease	5 (18.5)	3 (11.1)	2 (3.8)	10 (9.4)
Hepatic disease	4 (14.8)	2 (7.4)	2 (3.8)	8 (7.5)
Malignancy	1 (3.7)	3 (11.1)	1 (1.9)	5 (4.7)

Smoker	8 (29.6)	9 (33.3)	3 (5.8)	20 (18.9)

Functional status				
Ambulatory	20 (74)	25 (92.6)	52 (100)	97 (91.5)
Bed bound	7 (26)	2 (7.4)	0	9 (8.5)

Source of admission				
Emergency room	10 (37)	11 (40.7)	48 (92.3)	69 (65.1)
Ward (medical/surgical)	15 (55.6)	11 (40.7)	0	26 (24.5)
Operation room	0	4 (14.9)	0	4 (3.8)
Other hospitals	2 (7.4)	1 (3.7)	4 (7.7)	7 (6.6)

Admission diagnosis				
Head trauma	0	0	12 (23.1)	12 (11.3)
Polytrauma	0	0	39 (75)	39 (36.8)
Respiratory failure	11 (40.7)	3 (11.1)	0	14 (13.2)
Sepsis	4 (14.9)	8 (29.6)	0	12 (11.3)
Neurological disease	10 (37)	9 (33.3)	1 (1.9)	20 (18.9)
Cardiovascular disease	0	2 (7.4)	0	2 (1.9)
Intra-abdominal disease	1 (3.7)	4 (14.8)	0	5 (4.7)
Miscellaneous	1 (3.7)	1 (3.7)	0	2 (1.9)

SOFA^*∗*^ score (mean ± SD)	7.8 ± 3.0	5.7 ± 3.3	6.2 ±3.4	6.5 ± 3.4

Duration of mechanical ventilation before developing VAP (mean ± SD) (days)	17.0 ± 11.9	11.4 ± 9.8	9.5 ± 6.8	11.9 ± 9.5

Inappropriate antibiotic therapy	10 (37)	8 (29.6)	5 (9.6)	23 (21.7)

^*∗*^SOFA: Sequential Organ Failure Assessment.

**Table 2:** Antibiotic sensitivity pattern of bacteria isolated from respiratory specimen of patients with VAP.

Antibiotics	*Pseudomonas* *N* (%)	*Enterobacter* *N* (%)	*Klebsiella* *N* (%)	*E. coli* *N* (%)	*Acinetobacter* *N* (%)	*Stenotrophomonas* *N* (%)	*Haemophilus* *N* (%)	*Staphylococcus* *N* (%)	*Streptococcus* *N* (%)
Penicillin-G	—	—	—	—	—	—	—	7 (33.3%)	3 (60%)
Oxacillin	—	—	—	—	—	—	—	13 (61.9%)	—
Ampicillin	—	0 (0%)	0 (0%)	1 (20%)	—	—	15 (93.8%)	—	—
Amoxicillin/clavulanate	—	0 (0%)	16 (64%)	1 (20%)	—	—	16 (100%)	—	5 (100%)
Piperacillin/tazobactam	36 (92.3%)	17 (70.8%)	20 (80%)	4 (80%)	5 (21.7%)	—	—	—	—
Cefuroxime	—	2 (8.3%)	18 (72%)	1 (20%)	—	—	15 (93.8%)	—	—
Ceftriaxone	—	18 (75%)	18 (72%)	1 (20%)	—	—	—	—	5 (100%)
Ceftazidime	33 (84.6%)	—	—	1 (20%)	2 (8.7%)	—	—	—	—
Cefepime	34 (87.2%)	—	18 (72%)	1 (20%)	4 (17.4%)	—	—	—	—
Clindamycin	—	—	—	—	—	—	—	19 (90.5%)	5 (100%)
Erythromycin	—	—	—	—	—	—	—	17 (80.9%)	5 (100%)
Vancomycin	—	—	—	—	—	—	—	21 (100%)	—
Cotrimoxazole	—	24 (100%)	17 (68%)	1 (20%)	5 (21.7%)	4 (100%)	11 (68.8%)	—	—
Ciprofloxacin	36 (92.3%)	24 (100%)	24 (96%)	1 (20%)	5 (21.7%)	—	—	—	—
Levofloxacin	—	—	—	—	—	3 (75%)	—	—	—
Gentamicin	36 (92.3%)	24 (100%)	25 (100%)	2 (40%)	5 (21.7%)	—	—	—	—
Meropenem	36 (92.3%)	23 (95.8%)	25 (100%)	5 (100%)	5 (21.7%)	—	—	—	—
Colistin	39 (100%)	—	—	—	23 (100%)	—	—	—	—

*Total number of isolates*	*39*	*24*	*25*	*5*	*23*	*4*	*16*	*21*	*5*

**Table 3:** Association of epidemiological and clinical variables with outcome (*N* = 106).

Variable	Survivors	Nonsurvivors	*p* value
Age			
Mean ± SD (years)	43.1 ± 17.8	57.8 ± 16.8	0.001
<60 years	66 (84.6%)	12 (15.4%)	0.001
≥60 years	15 (53.6%)	13 (46.4%)	

Gender			
Male	69 (81.2%)	16 (18.8%)	0.02
Female	12 (57.1%)	9 (42.9%)	

Ethnicity			
Qatari	20 (64.5%)	11 (35.5%)	0.338
African	11 (78.6%)	3 (21.4%)	
Indian subcontinent	38 (80.9%)	9 (19.1%)	
Southeast Asians	5 (100%)	0 (0%)	
Others	7 (77.8%)	2 (22.2%)	

Number of comorbidities			
0	53 (91.4%)	5 (8.6%)	0.001
1	7 (53.8%)	6 (46.2%)	
≥2	21 (60%)	14 (40%)	

Comorbidities			
Diabetes mellitus	18 (60%)	12 (40%)	0.012
Without diabetes mellitus	63 (82.9%)	13 (17.1%)	
Hypertension	20 (55.6%)	16 (44.4%)	0.001
Without hypertension	61 (87.1%)	9 (12.9%)	
Cardiovascular disease	9 (60%)	6 (40%)	0.106
Without cardiovascular disease	72 (79.1%)	19 (20.9%)	
Renal disease	4 (57.1%)	3 (42.9%)	0.214
Without renal disease	77 (77.8%)	22 (22.2%)	
Neurological condition	4 (57.1%)	3 (42.9%)	0.214
Without neurological condition	77 (77.8%)	22 (22.2%)	
Respiratory disease	4 (40%)	6 (60%)	0.004
Without respiratory disease	77 (80.2%)	19 (19.8%)	
Hepatic disease	4 (50%)	4 (50%)	0.067
Without hepatic disease	77 (78.6%)	21 (21.4%)	
Malignancy	4 (80%)	1 (20%)	0.847
Without malignancy	77 (76.2%)	24 (23.8%)	

Associated factor			
Smoker	11 (55%)	9 (45%)	0.012
Nonsmoker	70 (81.4%)	16 (18.6%)	

Functional status			
Ambulatory	77 (79.4%)	20 (20.6%)	0.018
Bed bound	4 (44.4%)	5 (55.6%)	

Admitting ICU			
Medical ICU	13 (48.1%)	14 (51.9%)	0.001
Surgical ICU	19 (70.4%)	8 (29.6%)	
Trauma ICU	49 (94.2%)	3 (5.8%)	

Source of admission			
Emergency room	61 (88.4%)	8 (11.6%)	0.001
Ward (medical/surgical)	12 (46.2%)	14 (53.8%)	
Operating room	2 (50%)	2 (50%)	
Other hospitals	6 (85.7%)	1 (14.3%)	

Admission diagnosis			
Head trauma	12 (100%)	0 (0%)	0.001
Polytrauma	36 (92.3%)	3 (7.7%)	
Respiratory failure	6 (42.9%)	8 (57.1%)	
Sepsis	8 (66.7%)	4 (33.3%)	
Neurological disease	12 (60%)	8 (40%)	
Cardiovascular disease	1 (50%)	1 (50%)	
Intra-abdominal disease	5 (100%)	0 (0%)	
Miscellaneous	1 (50%)	1 (50%)	

SOFA score on admission (mean ± SD)	6.2 ± 3.2	7.5 ± 3.6	0.08

Duration of mechanical ventilation before developing VAP (days) (mean ± SD)	11.9 ± 9.7	12.0 ± 9.1	0.95

Onset of VAP			
Early (≤4 days)	16 (80%)	4 (20%)	0.675
Late (>4 days)	65 (75.6%)	21 (24.4%)	

Inappropriate antibiotic therapy	9 (11.3%)	14 (58.3%)	0.001
